# Perovskite B-Site Compositional Control of [110]_p_ Polar Displacement Coupling in an Ambient-Pressure-Stable Bismuth-based Ferroelectric**

**DOI:** 10.1002/ange.201203884

**Published:** 2012-09-28

**Authors:** Michelle R Dolgos, Umut Adem, Alicia Manjon-Sanz, Xinming Wan, Tim P Comyn, Timothy Stevenson, James Bennett, Andrew J Bell, T Thao Tran, P Shiv Halasyamani, John B Claridge, Matthew J Rosseinsky

**Affiliations:** M. R. Dolgos, U. Adem, A. Manjon-Sanz, X. Wan, J. B. Claridge, M. J. Rosseinsky Department of Chemistry, University of LiverpoolLiverpool, L69 7ZD (UK); T. P. Comyn, T. Stevenson, J. Bennett, A. J. Bell Institute for Materials Research, University of LeedsLeeds, LS2 9JT (UK); T. T. Tran, P. S. Halasyamani University of Houston, Department of ChemistryHouston, TX 77204 (USA)

**Keywords:** bismuth, diffraction techniques, ferroelectricity, perovskites, polar structures

Piezoelectrics are key functional materials in actuator and sensor applications.^1^ Current technology exploits lead-based materials displaying a morphotropic phase boundary (MPB) between two ferroelectric solid solutions of different symmetries and polarization directions. This is best exemplified by the PbZr_1−*x*_Ti_*x*_O_3_ (PZT) perovskite system where the distortions driving the piezo response are produced by the stereo-active electron lone pair of the Pb^2+^ cation on the A site of the ABO_3_ perovskite structure.^2^ Due to the environmental impact of lead, there is a considerable focus on the synthesis of lead-free electroceramics. It is not however clear if the complex crystal chemistry underlying the structural phase boundaries at the MPB in lead-based systems can be simply translated into lead-free analogues. In particular the balance between distortions driven by the A and B site cations and the role of octahedral tilting and tolerance factor considerations are expected to be quite different in non-lead systems, as Pb^2+^ is significantly larger than the more highly charged Bi^3+^ often considered as an alternative polarization-generating cation.^3^ However, the smaller size of bismuth ions generally requires high-pressure synthesis conditions to form a perovskite. Only a small group of known perovskites exist with A-site bismuth cations that are stable at ambient pressure, based on BiFeO_3_,^4^ Bi_2_Mn_4/3_Ni_2/3_O_6_,^5^ and Bi(Fe_2/8_Ti_3/8_Mg_3/8_)O_3_ (BFTM).^6^ Bi_2_Mn_4/3_Ni_2/3_O_6_ is antiferrodisplacive,^5^ while both BiFeO_3_ and BFTM adopt the R′ *R*3*c* structure where the [111]_p_ displacements of untilted R (space group *R*3*m* in the PZT case) are coupled with octahedral rotation about the same axis (the prime symbol denotes the presence of tilts). In order to access MPBs, polar structures with distinct polarization directions away from [111]_p_ in these Bi-based families are required.

We investigated the pseudoternary phase field BFTM–LaFeO_3_ (LFO)–La_2_MgTiO_6_ (LMT) where the introduction of LFO (tolerance factor *t*=0.95) would alter the average structural asymmetry on the A site by combining the aspherical Bi^3+^ and spherical La^3+^ cations, and the addition of Mg^2+^ and Ti^4+^ from LMT (*t*=0.95) on the B site would reduce the dielectric loss caused by the increase in Fe^3+^ content. LFO adopts the *Pmnb* structure of GdFeO_3_ with an a^+^b^−^b^−^ tilt system and 0.18 Å antiferrodistortive La^3+^ displacements along the [110]_p_ direction. LMT (*P*2_1_/*a*) has the same A-site displacement pattern and tilt system plus rock salt ordered B-site cations.^7^

Phases in the BFTM–LMT–LFO (BLFTM) field (Figure 1 a) were prepared according to the protocol set out in Section 1 of the Supporting Information. Four distinct classes of powder diffraction patterns are observed. BFTM (*t*=0.96) is the only material with the rhombohedral *R*3*c* space group, and is surrounded by a two-phase region. At low LFO content and along the BFTM–LMT line, an orthorhombic perovskite phase and an Aurivillius phase coexist (XRD data Figure S1, Supporting Information). However, single phases emerge at higher substitution levels. These phases adopt the nonpolar *Pmnb* structure at high LFO/LMT content, but a new orthorhombic phase emerges between the *Pmnb* and multiphase regions, as shown by the evolution of the lattice parameters along the (1−*x*)BFTM–*x*LFO line in Figure 1 b. Powder second harmonic generation (SHG) measurements^8^ show that the non-*Pmnb* BLFTM samples are non-centric (*x*=0.28, 0.37, 0.40, 0.42), whereas the *Pmnb* materials (*x*=0.5, 0.67) are centric as expected. Thin films can be grown by pulsed laser deposition (Section 5, Supporting Information).

**Figure 1:**
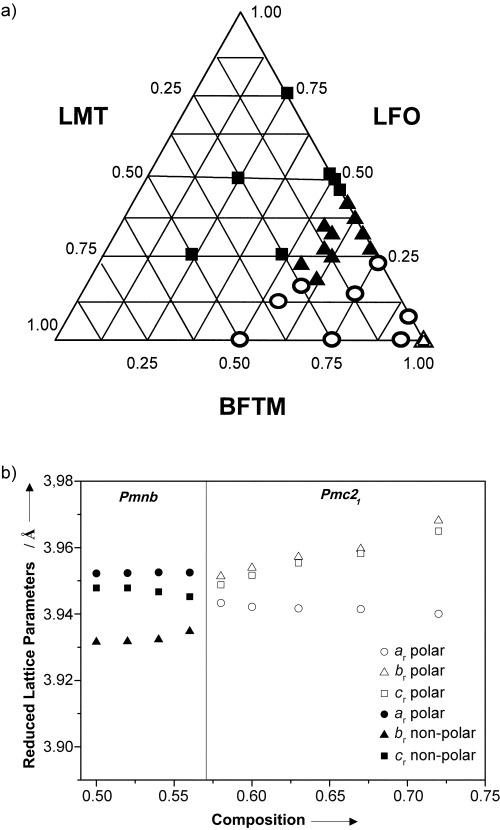
a) Phase diagram of the Bi(Fe_2/8_Ti_3/8_Mg_3/8_)O_3_–LaFeO_3_–La(Mg_1/2_Ti_1/2_)O_3_ BLFTM system showing the four distinct categories of phase assemblage found in the system (▪ *Pmnb*; ▴ *Pmc*2_1_; ○ two-phase mixture; ▵ *R*3*c*) and b) evolution of the reduced lattice parameters along the BFTM–LFO line expressed as the composition of BFTM. Filled and open symbols correspond to materials shown to be centric and non-centric, respectively, by second harmonic generation measurements.

The structural characterization of the new non-centric BLFTM phase will focus on the Bi_0.72_La_0.28_(Fe_0.46_Ti_0.27_Mg_0.27_)O_3_ (0.72BFTM–0.28LFO) composition. Of the candidate space groups which gave similar model-independent Lebail fits in the observed 2*a_p_*× 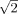
*a_p_*× 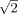
*a_p_* cell only *Pmnb* and *Pmc*2_1_ give acceptable Rietveld refinements of neutron (HRPD, GEM) (Figure 2 a) and synchrotron X-ray (ID31) data. The *Pmc*2_1_ refinement is superior (*R*_wp_=7.19 % versus 8.25 %), with the non-polar *Pmnb* model unable to satisfactorily fit the (110), (200), and (011) reflections (Figure 2 b).

**Figure 2:**
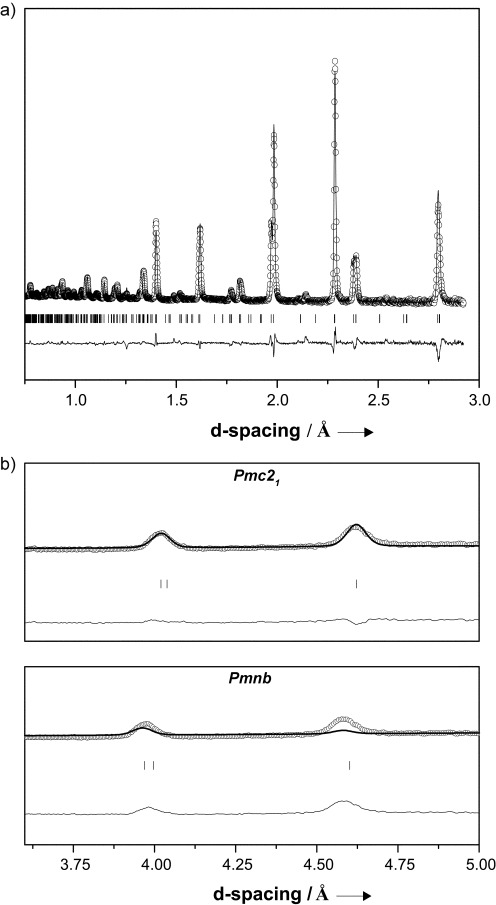
Rietveld refinement of a) neutron diffraction data (HRPD) for 0.72BFTM–0.28 LFO in space group *Pmc*2_1_. b) Comparison of the fits in *Pmc*2_1_ (top) and *Pmnb* (bottom) for the (110), (200), and (011) reflections in 0.72BFTM–0.28LFO from the GEM neutron diffraction data. The circles represent the observed data, the thick line represents the model and the difference can be seen below.

The *Pmc*2_1_ structure of 0.72BFTM–0.28LFO (Section 3.1, Supporting Information) is adopted at low temperature by the high-pressure form of the B-site-driven CdTiO_3_ ferroelectric,^9^ (though more recent neutron diffraction studies have cast doubt on this^10^), the high field form of NaNbO_3_^11^ and sol–gel synthesized NaNbO_3_.^12^ There are one B site and two A sites in the asymmetric unit, with the Rietveld refinement showing no indication of Bi/La cation ordering. The two A sites are arranged in alternating layers along *a* (Figure 3 a and b). Each A site has a ferroelectric displacement with a distinct amplitude (0.31 Å A1 and 0.17 Å A2) along the *z* ([110]_p_) direction (Figure 3 c), and distinct Bi–O environments (Figure 3 e,f). The B site (Figure 3 g) also has a 0.14 Å ferroelectric displacement along [110]_p_. There are also small (<0.05 Å) antiferrodistortive displacements at both A sites along *b* and *c* and at the B site along *a* (Figure 3 d). The ionic polarization calculated^13^ from the refined structure is 30.6 μC cm^−2^.

**Figure 3:**
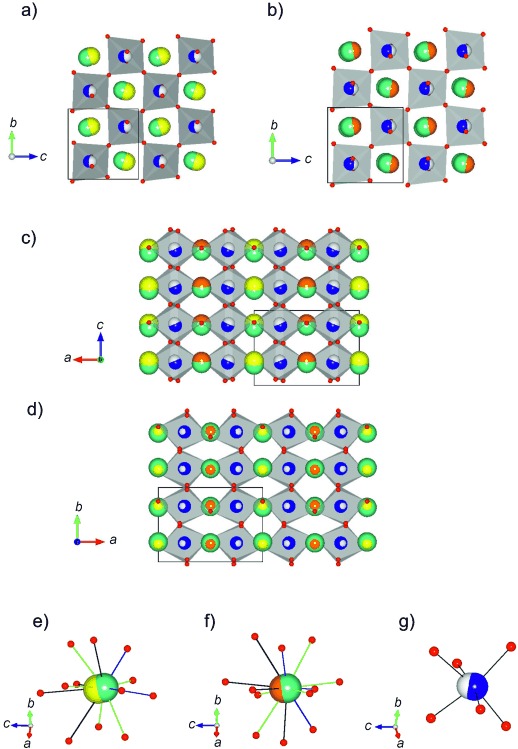
*Pmc*2_1_ room temperature structure of 0.72BFTM–0.28LFO. A-site cations represented in green, B-site cations blue, and oxygen atoms in red. The centroids of the oxide anions coordinating the A1 site are shown in yellow, A2 centroids orange, B centroids white. Viewed along the *a* direction, with a) the A1 layer on top and b) the A2 layer on top. c) Viewed along *b* showing the FE displacements of A1, A2, and B along the *c* direction. d) Viewed along *c* showing the A1 and A2 AFE displacements along *b* and the B-site AFE displacements along *a*. e) Coordination environment of the A1 cation. There are four short Bi–O contacts (blue) four intermediate (green) and four long bonds (black). Displacement components (0, −0.09 Å, 0.31 Å) in the unit cell reference frame. f) Coordination environment of the A2 cation. This site has three short contacts (blue) and nine which are less distinctly separated than for A1, divided here into six bonds between 2.6 and 3 Å (green) and three bonds longer than 3 Å (black). Displacement components (0, −0.03 Å, 0.17 Å). g) B-site octahedral environment. Displacement components: (−0.06 Å, 0.009 Å, 0.14 Å).

Measurement of the synchrotron X-ray and neutron powder diffraction patterns as a function of temperature reveals a structural phase transition from *Pmc*2_1_ to *Pmnb* above 650±5 °C (Figure S6). This structure (Section 3.2, Supporting Information) maintains the a^+^b^−^b^−^ tilt system of *Pmc*2_1_. There is only one crystallographically distinct A site, which is displaced 0.08 Å antiferrodistortively along [110]_p_ and no B-site displacement.

The atomic displacements for the polar *Pmc*2_1_ BLFTM structure and the high-temperature (HT) *Pmnb* phase can be understood in terms of distortion modes derived from the 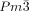
*m a_p_* aristotype cell, evaluated with the Isodisplace^14^ and Amplimodes^15^ programs (for further details and mode amplitudes see Section 3.3, Supporting Information). *Pmnb* (basis={(2,0,0),(0,1,−1)(0,1,1)}, origin=(0,0,0)) is produced by the R_4_^+^ (Figure 4 b) antiphase a^0^b^−^b^−^ and in-phase M_3_^+^ (Figure S8a) a^+^b^0^b^0^ octahedral tilts which are the most significant modes. The associated antiferrodistortive A-site displacements along *c*, 0.08 Å from the centroids of their coordinating oxygens, are from the X_5_^+^ mode^16^ (Figure 4 c), giving the observed single A-site structure. The *Pmc*2_1_ phase (basis={(2,0,0),(0,1,−1)(0,1,1)}, origin=(1/2,1/2,0)) is driven by the onset of the dominant Γ_4_^−^ polar displacement at the A and B sites (Figure 4 a). This ferroelectric displacement is along [110]_p_, as in the *Amm*2 (O) phase of BaTiO_3_^17^ and the high-field form of PbZrO_3_.^18^ In-phase combination of this ca. 0.22 Å magnitude polar displacement with the 0.08 Å antiferrodistortive displacement (X_5_^+^ mode) present in the *Pmnb* precursor affords the 0.31 Å displacement at the A1 site, while the out-of-phase combination gives the 0.14 Å polar displacement at the A2 site along the [110]_p_ direction. The small A-site antiferrodisplacive R_5_^+^ mode (Figure S8b) along *b* is unchanged between *Pmnb* and *Pmc*2_1_. There is a single B site in *Pmc*2_1_ with the Γ_4_^−^-driven polar displacement along *c* accompanied by a smaller antiferroelectric displacement along *a* from the X_3_^−^ mode (Figure 4 d). Only one B site results because the ferroelectric and antiferroelectric displacement directions are perpendicular.

**Figure 4:**
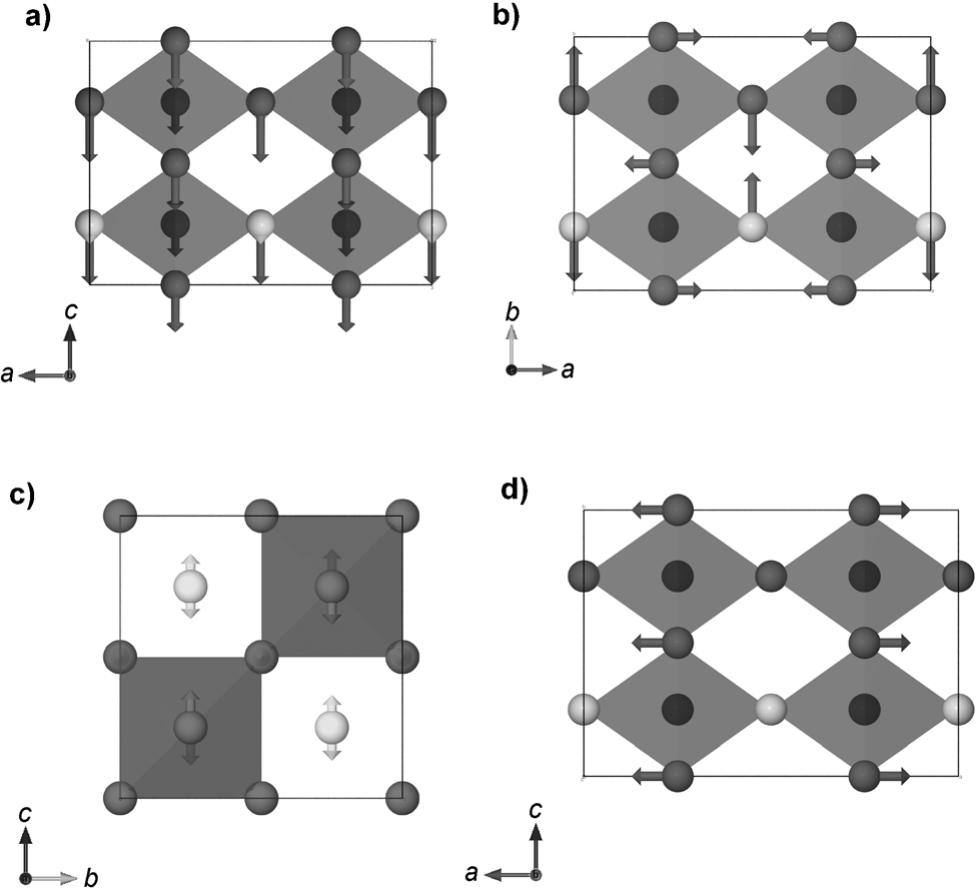
Important displacement modes in the *Pmc*2_1_ and *Pmnb* structures of BLFTM a) Γ_4_^−^ ferroelectric displacement mode in *Pmc*2_1_, b) R_4_^+^ out-of-phase tilting mode (both structures), c) the X_5_^+^ mode which is responsible for A-site antiferrodistortive displacements along *c* in both structures, and d) the X_3_^−^ mode with antiferrodistortive B-site displacements along *a* in *Pmc*2_1_. A-site cations represented in white, B-site cations black, and oxygen atoms in gray.

Electrical properties of BLFTM were measured on 97+% dense ceramics, with dielectric loss reduced by the addition of 0.2 wt % MnO_2_,^19^ which lowers tan *δ* from 0.036 to 0.025 at 1 kHz and from 0.589 to 0.034 at 1 Hz for 0.72BFTM–0.28 LFO. The low-frequency loss (Figure 5 a) is comparable to other shared A-site Bi-based ferroelectrics for example, Na_0.5_Bi_0.5_TiO_3_ (>0.05 at 1 kHz).^20^ 0.625BFTM–0.25LFO–0.125LMT has the lowest tan *δ*, suggesting that LMT is particularly effective in reducing the loss. All the *Pmc*2_1_ [110]_p_ displaced materials have higher permittivities than [111]_p_ displaced rhombohedral BFTM, and the broad step-like decrease in permittivity for BFTM above 1 kHz, suggestive of a relaxation phenomenon, is not observed in the new polar phase. The magnitude of the relative permittivity rules out the possibility of contributions from extrinsic charge carriers at grain boundaries or electrode–sample interfaces.^21^

**Figure 5:**
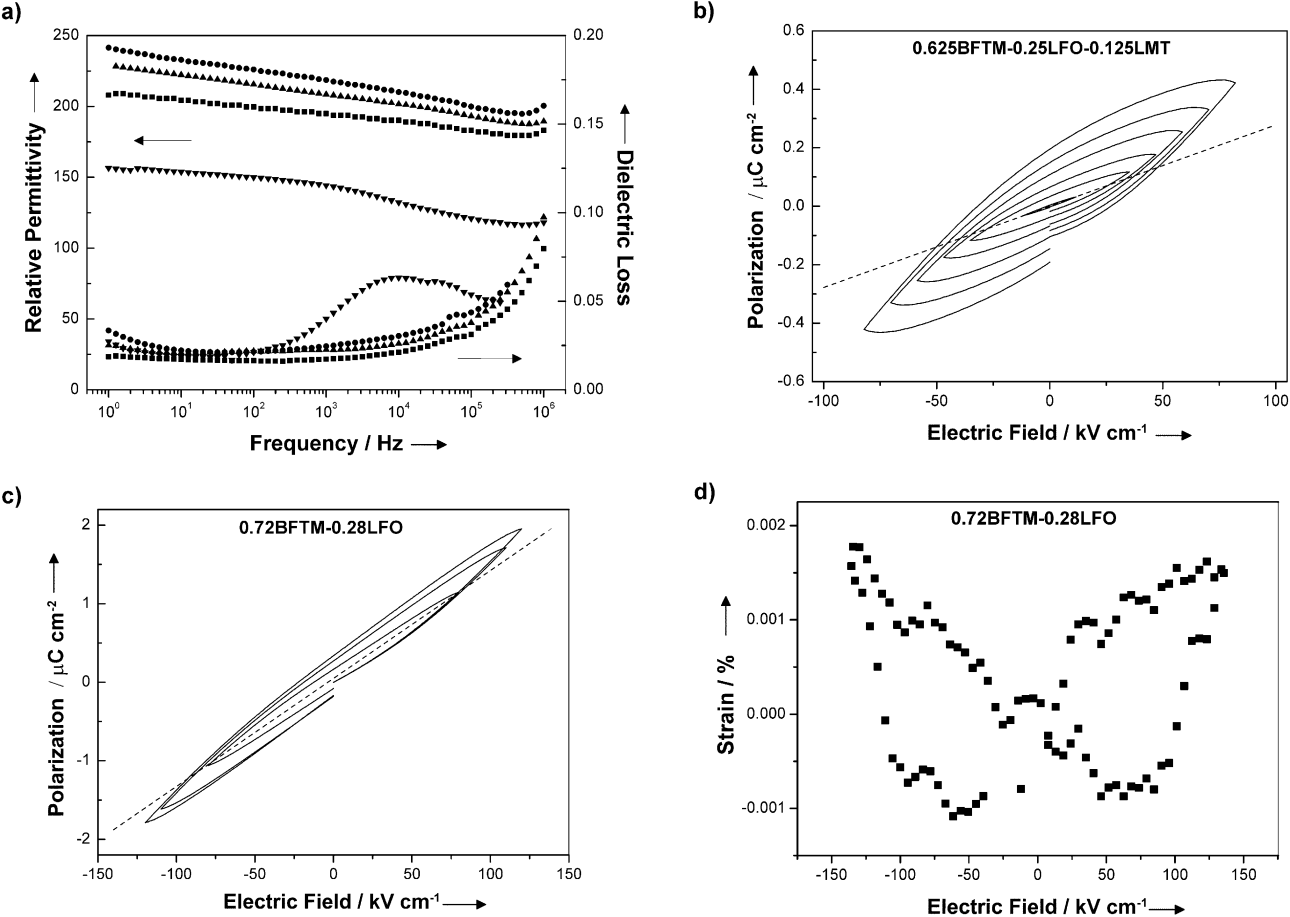
a) Room temperature permittivity and loss as a function of frequency of BFTM (▾), 0.72BFTM–0.28LFO (•), 0.625BFTM–0.25LFO–0.125LMT (▪), and 0.58BFTM–0.42LFO (▴). b) *P*(*E*) loop for 0.625BFTM–0.25LFO–0.125LMT, c) *P*(*E*) loop for 0.72BFTM–0.28LFO corresponding to d) the strain-field loop of the same composition. The dotted line in (b) and (c) represent the slope of the *P*(*E*) loop at the lowest voltage and is used to demonstrate nonlinearity.

The polarity shown in the structure and SHG measurements was confirmed by measurements of piezoelectric behavior. Strain-field measurement on *Pmc*2_1_ 0.72BFTM–0.28LFO (Figure 5 d) shows a butterfly loop, with a negative strain component. This negative strain is related to domain back-switching during the bipolar cycles and demonstrates typical ferroelectric behavior.^22^ The coercive field, calculated from the minimum in the strain field plot, is equal to 62 kV cm^−1^. The calculated strain is 0.003 % and the high-field *d*_33_ value obtained from the strain-field measurements is 1.68 pC N^−1^. The measured piezoelectric coefficient is 0.25 pC N^−1^.

It was not possible to obtain a saturated *P*(*E*) (*P*=polarization, *E*=electric field) hysteresis loop before dielectric breakdown, which is a common problem in BiFeO_3_ ceramics.^23^ This can be attributed to a high Curie temperature and coercive field. The best *P*(*E*) loop came from the *Pmc*2_1_ structure composition 0.625BFTM–0.25LFO–0.125LMT (Figure 5 b). Although the loop is not saturated, electrical polarization does not evolve as a linear function of the driving-field amplitude indicating nonlinearity (a necessity for ferroelectrics) and therefore domain wall motion.^24^ *P*(*E*) for 0.72BFTM–0.28LFO (Figure 5 c) is not saturated but shows non-linearity, indicating the presence of domain wall motion, consistent with the strain-field measurement on this composition.

Figure 6 represents the A-site displacements in polar structures derived from the ideal perovskite, with the effect of octahedral tilting in systems containing smaller A cations than Pb^2+^ evident. The O′ *Pmc*2_1_ structure found here corresponds to the polarization direction changing from [111]_p_ in the R′ parent phase BFTM to [110]_p_, driven by the substitution of La^3+^ onto the A site, because of the tilts and displacements in this direction in LaFeO_3_. La^3+^ substitution into R′ BiFeO_3_ also induces [110]_p_ A- and B-site displacements, but in this case the displacements are coupled *anti*ferrodistortively to produce the antiferroelectric PbZrO_3_ structure (Figure 6; O_A_′ denotes a tilted antiferroelectric structure).^25^ The striking difference in the coupling of the [110]_p_ displacements in the present BLFTM case may arise from the presence of Mg^2+^ and particularly Ti^4+^ on the B site in the BFTM parent, as Ti^4+^ produces ferroelectric [110]_p_ displacements in O BaTiO_3_.^17^ The O′ structure features two A sites with distinct polarizations because of the competition between La-driven [110]_p_ antiferrodisplacive motions and the dominant ferroelectric displacements along the same direction driven by Bi^3+^ and Ti^4+^, reflecting structural frustration between the bonding preferences of the two A-site cations. In the present BLFTM system, the O′ structure is separated by two-phase regions involving non-perovskite impurities from the R′ structure, and bridging this phase gap is important in the search for enhanced functionality associated with potential phase boundaries differing in polarization direction.

**Figure 6:**
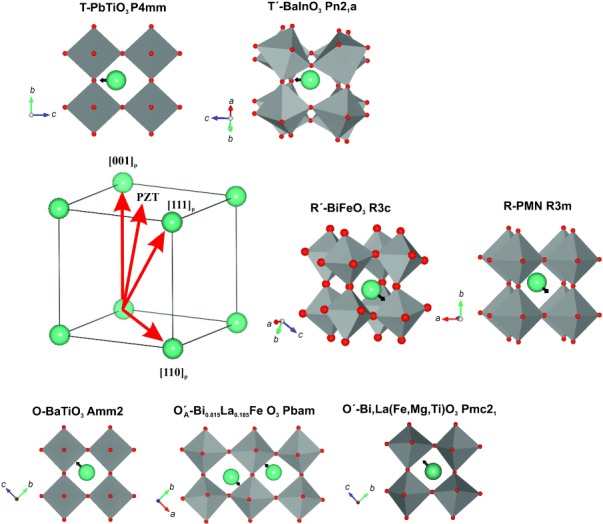
Examples of perovskites with A-site ferroelectric displacements along the [001]_p_, [110]_p_, and [111]_p_ directions in relation to the ideal cubic perovskite *a_p_* cell. The tilted T′ structure of BiInO_3_ is only accessible at high pressure:^26^ only the FE displacements are shown as the bismuth ion has an AFE component as well. The polar O′ structure observed for BLFTM in the present study contrasts with the antiferrodistortive coupling of [110]_p_ displacements in the PbZrO_3_ structure of La-substituted R′ BiFeO_3_. The arrow representing the displacements in M-PZT indicates a Pb^2+^ displacement mainly along [100]_p_, but with a significant component along [110]_p_.

## Supplementary material

Detailed facts of importance to specialist readers are published as ”Supporting Information”. Such documents are peer-reviewed, but not copy-edited or typeset. They are made available as submitted by the authors.
