# Supplementary material for: Perovskite B-Site Compositional Control of [110]p Polar Displacement Coupling in an Ambient-Pressure-Stable Bismuth-based Ferroelectric
Source: Angew Chem Weinheim Bergstr Ger. 2012 Sep 28;124(43):10928–33. doi: 10.1002/ange.201203884 (PMC4373144; doi:10.1002/ange.201203884)
Supplement: Supplementary file 1 [file ange0124-10928-SD1.pdf]

Angewandte Chemie
Eine Zeitschrift der Gesellschaft Deutscher Chemiker

Supporting Information

© Wiley-VCH 2012

69451 Weinheim, Germany

Perovskite B-Site Compositional Control of $[110]_p$ Polar Displacement Coupling in an Ambient-Pressure-Stable Bismuth-based Ferroelectric**

Michelle R. Dolgos, Umut Adem, Alicia Manjon-Sanz, Xinming Wan, Tim P. Comyn, Timothy Stevenson, James Bennett, Andrew J. Bell, T. Thao Tran, P. Shiv Halasyamani, John B. Claridge, and Matthew J. Rosseinsky**

ange_201203884_sm_miscellaneous_information.pdf

Supplementary Information

1. Experimental

Starting materials of Bi_2O_3 (Alfa Aesar, 99.9995%), La_2O_3 (Alfa Aesar 99.999%; heated to 950°C for several hours before use), Fe_2O_3 (Alfa Aesar, 99.998), TiO_2 (Alfa Aesar, 99.995%), and $4MgCO_3{\cdot}Mg(OH)_2{\cdot}4H_2O$ (Aldrich 99.99%) were mixed in stoichiometric amounts and milled in a planetary mill with 8 yttria stabilized zirconia balls (10 mm diameter) and 10 mL of ethanol for 45 cycles with each cycle consisting of 15 minutes forward, 10 minutes rest, and 15 minutes backwards at a rate of 350 rpm. The resulting homogenous mixture was pressed into pellets and placed in an alumina crucible lined with Pt foil on the bottom to prevent reaction with the alumina while heating. The pellets were then fired in air at 950°C for 14 hours to form a phase pure powder.

For physical measurements, the powder was milled as above but with an addition of 0.2 wt% MnO_2 and 2 wt% Polyvinyl Butyral binder. Pellets were pressed using either a 10 mm or 8mm die then the pellets were sealed within an evacuated latex bag and pressed using a cold isostatic press at 2000 bar. The pellets were sintered at 975 °C 4hr; 950 °C for 15 hours in an O_2 atmosphere to obtain a density of > 95%. The density was measured using an Archimedes balance. Electrodes for the ferroelectric and electrostriction measurements were either gold sputtered or painted with silver.

Synchrotron powder X-ray diffraction data were collected at ESRF on the ID31 beamline with λ =0.39986 Å. Data was collected over a 2θ range from 2° - 44° at room temperature in a quartz capillary. Variable temperature data were also collected scanning the temperature range of 30 °C to 650 °C with a long scan at 650 °C to be used for a Rietveld refinement. Neutron data were collected at the ISIS spallation neutron source at Rutherford Appleton Laboratory on the HRPD beam line and on the GEM beamline. High temperature

neutron data were collected at 650°C on HRPD to 120 μA with continuous scans to 20 μA upon heating and cooling. Data collected on the backscattering banks were used in the refinement. Topas Academic was used to index the powder XRD data.[1] All refinements were performed using the software program GSAS/EXPGUI and Topas Academic.[2]

The refinements showed no anisotropic broadening and were fit using a pseudo-Voight profile. In order for the refinement to remain stable, the Bi/La cations were constrained to have the same atomic positions and thermal parameters, with the same constraints being placed on the Ti/Mg/Fe B cations. Attempts to separate the positions resulted in a divergence of the refinement.

Energy dispersive spectroscopy data were collected with a JEOL 2000FX electron microscope and showed good agreement with target compositions. The samples were prepared by grinding the powder in acetone and the small crystallites in suspension were deposited onto a carbon file supported by a copper grid. Electron diffraction data were also collected with the same microscope.

Impedance measurements were performed by using a Solartron 1255B Frequency Response Analyzer and a Solartron 1296 dielectric interface. Measurements were taken over the frequency range of 1Hz to 1MHz at room temperature. Ferroelectric measurements were performed on a thin pellet (<0.4mm thickness) with a Radiant Precision high voltage interface and a T Rek 609B high voltage amplifier were used to measure the response as a function of voltage, at a frequency of 1 Hz. The electric field was increased in a stepwise manner until dielectric breakdown. High-field piezoelectric measurements were performed using a Fotonic sensor MTI-2100 combined with a commercial Radiant Ferroelectric Tester Precision LC, in line with a Trek 5/80 high voltage amplifier, and Radiant high voltage interface using a frequency of 0.1Hz, increasing the electric field until dielectric breakdown occurred. The piezoelectric coefficient d_{33} was measured with a conventional Berlincourt

meter (Piezotest PM300, London, UK), by applying an alternating force of 0.25 N and a frequency of 110 Hz. Prior to measurement, the samples were poled in silicon oil at 200ºC. The voltage was increased slowly to up to 200 kV/cm and left for 20 minutes. Then the sample was slowly cooled to room temperature with the applied electric field before removing from the silicon oil, taking approximately three hours.

2. Elemental Analysis

Initial synthesis attempts targeted the composition $Bi_{0.75}La_{0.25}(Fe_{0.44}Ti_{0.28}Mg_{0.28})O_3$, which is 0.75BFTM + 0.25 LFO. However, X-ray diffraction revealed impurities >5% of an Aurivillius type phase. EDS results showed that the average of the actual composition was $Bi_{0.72}La_{0.28}(Fe_{0.53}Ti_{0.26}Mg_{0.21})O_3$.

The B-site compositions from the EDS may not be extremely accurate because Mg is at the very lower limit for the instrument. Therefore, to maintain a charge balance, a solid solution of 0.72BFTM-0.28$LaFeO_3$, which gave a composition of $Bi_{0.72}La_{0.28}(Mg_{0.27}Ti_{0.27}Fe_{0.46})O_3$ was then made. This resulted in a single phase perovskite from the XRD analysis. EDS analysis of 12 grains were examined to determine homogeneity. The expected ratio, normalized to Ti was Bi: 2.67, La: 1.04, Fe: 1.7, Ti: 1, Mg: 1. The average and standard deviation from EDX was found to be Bi: 2.67(1), La: 1.04(6), Fe: 1.89(19), Mg: 1.02(8) and Ti: 1.0.

3. Structural Characterization

3.1 Room Temperature phase

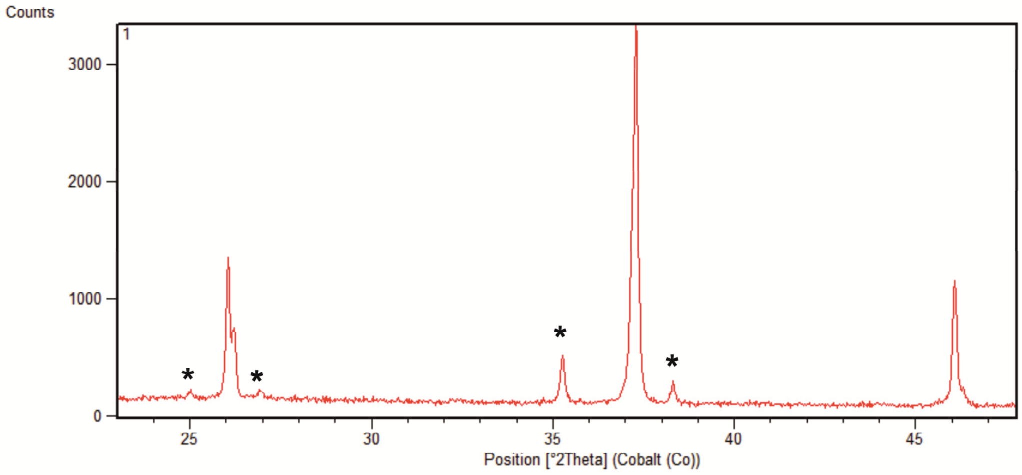

Figure S1: Example of an XRD pattern from the 2-phase region of the BLFTM phase diagram. This is the XRD pattern for 0.5BFTM-0.25LFO-0.25LMT. The main peaks are from an orthorhombic perovskite phase while the peaks denoted with asterisks are from an Aurivillius type phase. Lebail fits were performed with Topas Academic to determine the lattice parameters for all the phases involved.

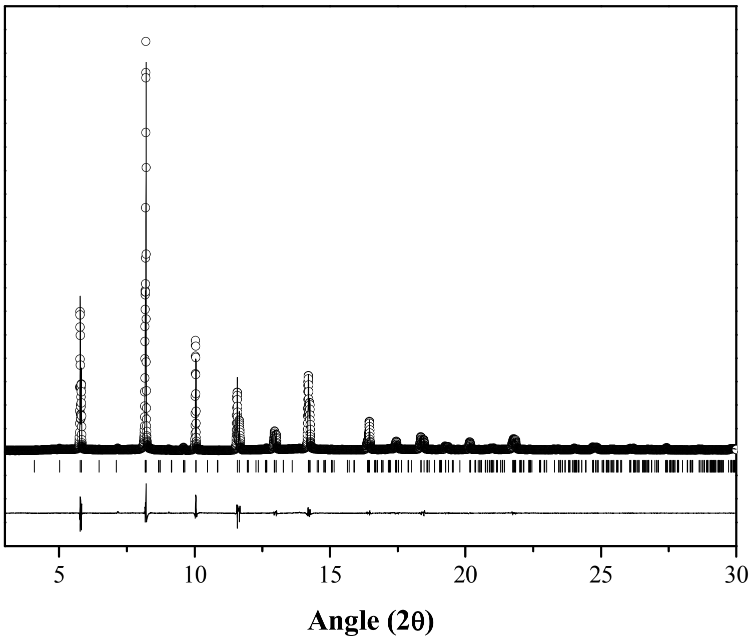

Figure S2: Rietveld refinement of synchrotron XRD data (ID31).

Table S1: Lattice parameters for all BLFTM compositions in the polar *$Pmc2_1$* phase

Composition	a / Å	b / Å	c / Å	Cell Volume / A^3
0.72BFTM- 0.28LFO	7.88014(9)	5.6118(1)	5.6073(2)	247.96(2)
0.67BFTM -0.33LFO	7.8830(1)	5.5997(4)	5.5978(5)	247.10(3)
0.63BFTM - 0.37LFO	7.8834(6)	5.5963(9)	5.5938(9)	246.81(6)
0.60BFTM - 0.40LFO	7.8843(2)	5.5917(6)	5.5885(7)	246.35(4)
0.58BFTM -0.42LFO	7.89761(8)	5.57669(9)	5.5692(1)	245.281(6)
0.625BFTM-0.25LFO -0.125LMT	7.8867(2)	5.5974(1)	5.5955(2)	247.01(1)
0.58BFTM-0.23LFO -0.19LMT	7.90590(8)	5.58293(9)	5.5730(1)	245.981(7)
0.58 BFTM- 0.33LFO -0.09LMT	7.90209(9)	5.58071(8)	5.5727(1)	245.754(7)
0.625BFTM- 0.1875LFO-0.1875 LMT	7.8856(2)	5.5933(6)	5.5948(6)	246.76(4)

Table S2: Lattice parameters for all BLFTM compositions in the nonpolar *Pmnb* phase

Composition	a / Å	b / Å	c / Å	Cell Volume / $Å^3$
0.56BFTM -0.44LFO	7.9050(4)	5.5794(3)	5.5646(4)	245.43(2)
0.54BFTM-0.46LFO	7.9051(2)	5.5815(2)	5.5611(2)	245.37(1)
0.52BFTM-0.48LFO	7.9047(2)	5.5803(2)	5.5603(2)	245.26(1)
0.5BFTM-0.5LFO	7.8945(3)	5.5831(2)	5.5659(2)	245.32(1)
0.33BFTM-0.67LFO	7.8869(4)	5.5732(2)	5.5595(1)	244.37(1)
0.25BFTM-0.75LFO	7.8869(4)	5.5703(2)	5.5597(2)	244.25(2)
0.25BFTM-0.5LFO- 0.25LMT	7.866(1)	5.5794(8)	5.5620(8)	244.11(6)
0.25BFTM- 0.25LFO -0.5LMT	7.883(3)	5.572(2)	5.564(2)	244.4(1)

Combined X-ray and neutron diffraction refinements were performed on 0.72BFTM – 0.28LFO using HRPD and ESRF data. Indexing was performed with Topas Academic software. The following figures are fits from *Pn2₁a* and *Pnm2₁*, the other subgroups of *Pmnb*.

Figure S3: Fits for the RT Rietveld refinements of 0.72BFTM-0.28LFO using the *Pnm2₁* and *Pn2₁a* space groups. The observed data is black, the model red, and the difference is in blue.

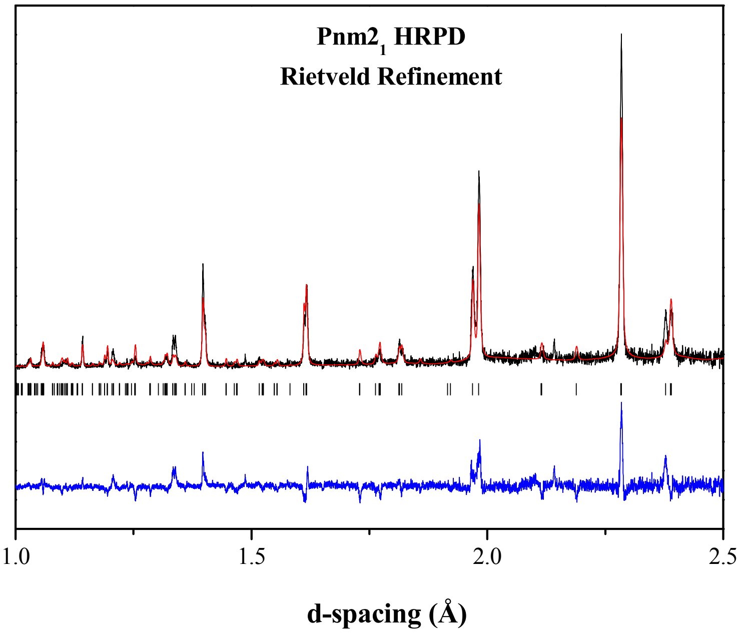

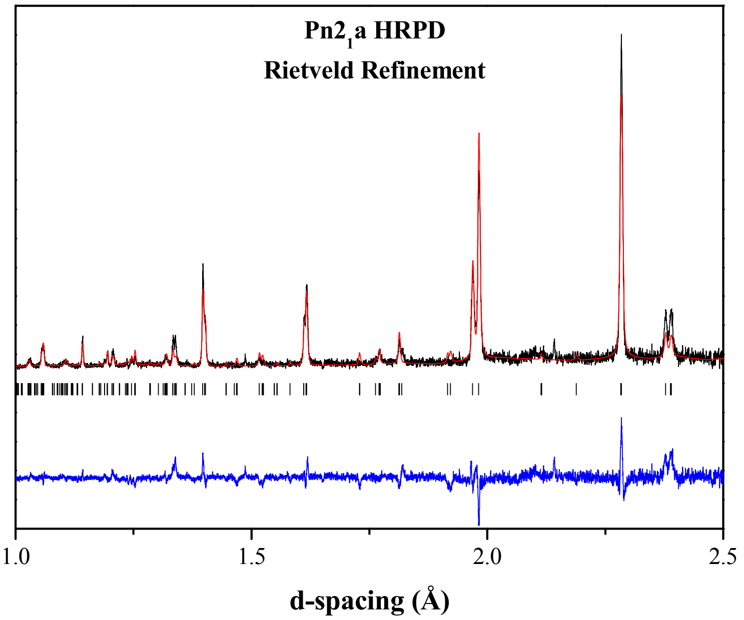

Table S3: Rietveld refinement goodness of fit results from the combined RT Rietveld refinement of 0.72BFTM – 0.28LFO HRPD and ID31 ESRF data.

Space group	R_{wp}
Pnma	9.66
$Pmc2_1$	8.85
$Pn2_1a$	12.26
$Pnm2_1$	13.45
Pc	12.22
P1m1	11.88
Pm11	10.41
P11m	12.71
$P2_111$	11.02
$P12_11$	11.82
$P112_1$	10.70
I4/m	13.51

Table S4: Atomic positions from RT Rietveld refinement of 0.72BFTM-0.28LFO in the *$Pmc2_1$* space group at room temperature.

Atom	x	y	z	Occupancy	B / Å^2
Bi1/La1	0	0.2687(7)	0.7716(8)	0.72/0.28	0.031(2)
Bi2/La2	0.5	0.2523(7)	0.7844(7)	0.72/0.28	0.0213(7)
Fe/Mg/Ti	0.749(2)	0.749(2)	0.8003(5)	0.46/0.27/0.27	0.0103(2)
O1	0	0.185(2)	0.349(1)	1	0.019(2)
O2	0.5	0.320(1)	0.309(2)	1	0.018(2)
O3	0.211(2)	0.519(3)	0.070(1)	1	0.029(2)
O4	0.287(2)	1.021(3)	0.569(2)	1	0.027(2)

Table S5: Bond lengths and angles from Rietveld refinement of 0.72BFTM-0.28LFO in the *$Pmc2_1$* space group at room temperature.

**Bond**	**Bond Length / Å**
A1 - O	2 x 2.33565(2)<br>2.4165(4)<br>2.57930(4)<br>2 x 2.74414(3)<br>2 x 2.88557(2)<br>3.09205(5)<br>3.23861(2)<br>2 x 3.25222(4)
A2 - O	2.40020(2)<br>2 x 2.44001(2)<br>2.69370(5)<br>2 x 2.77632(2)<br>2x2.87551(2)<br>2 .96719(6)<br>2 x 3.15911(3)<br>3.20526(5)
B-O	1.99749(2)<br>2.00311(3)<br>2.00622(3)<br>2.01259(3)<br>2.02142(3)<br>2.03123(2)
**Atoms**	**Angle / °**
O-Bi1/La1/-O	2 x 53.667(1), 2x54.126(1), 2x54.564(2), 2x54.629(1), 2x57.975(1), 2x59.737(2), 2x61.355(2), 2x61.490(3), 2x64.147(1), 2x65.948(2), 2x66.623(2), 2x67.92(1), 72.118, 74.474(2), 2x88.446(1), 88.518(1), 90.145(3), 2x90.340(1), 2x90.566(1), 2x90.829(2), 103.399(2), 2x108.617(2), 109.219(1), 2x111.389(1), 2x113.722(3), 2x114.038(1), 2x115.003(2), 2x117.720(1), 2x120.575(3), 2x122.035(1), 2x123.450(2), 2x125.656(1), 2x127.870(1), 2x134.202(1), 160.636(2), 162.263(1), 2x163.664(1), 2x178.002(3)
O-Bi2/La2-O	2x54.754(1), 2x55.285(2), 2x55.695(1), 2x59.123(1), 2x59.270(3), 2x59.851(1), 2x59.953(2), 2x61.559(1), 2x62.358(1), 2x65.003(2), 2x65.147(1), 2x65.502(1), 74.024(2), 79.329(2), 85.193(1), 86.698(2), 2x90.523(2), 2x90.564(1), 92.111(3), 94.888(1), 100.590(2), 2x104.771(1), 2x110.923(2), 2x113.771(1), 2x113.821(1), 2x115.792(2), 2x116.837(1), 2x117.564(1), 2x121.235(3), 2x123.990(1), 2x124.092(1), 2x124.834(1), 2x125.051(2), 2x130.432(2), 2x164.481(1), 164.522(1), 174.217(1), 2x176.043(2)
O-Fe/Mg/Ti-O	80.853(1), 81.755(1), 84.181(3), 87.412(2). 88.474(1), 89.308(1), 91.298(2), 91.451(3), 95.414(1), 96.289(1), 101.175(3), 168.292(2), 168.585(2), 168.936(1)

Table S6: Bond Valence Sums from Rietveld refinement of 0.72BFTM-0.28LFO in *$Pmc2_1$* at room temperature. Evidence of local strain in both the A-sites and the B-site can be found in the bond valence sums. In the A1 site, the La atom is over bonded while bismuth is under bonded. On the B-site the Ti is under-bonded and the Mg is over-bonded.

**Atom**	**BVS**
Bi1	2.6
La1	3.2
Bi2	2.7
La2	3.3
Fe	3.0
Ti	3.4
Mg	2.5

Table S7: Tolerance Factors for BFTM-LFO solid solutions. An ionic radius of 1.36 Å was used for a 12-coordinate Bi^{3+} ion. This demonstrates the evolution of the structural phase transition without a significant change in the tolerance factor, suggesting that the difference in preferred local symmetries between La^{3+} and Bi^{3+} are more significant.

**Composition**	**Tolerance Factor**	**Space Group**
BFTM	0.948249	R3c
0.72 BFTM-0.28 LFO	0.948249	$Pmc2_1$
0.67 BFTM-0.33 LFO	0.949763	$Pmc2_1$
0.5 BFTM-0.5 LFO	0.950269	Pmnb
0.33 BFTM-0.67 LFO	0.952298	Pmnb
0.25 BFTM-0.75 LFO	0.952806	Pmnb
LFO	0.954335	Pmnb

Table S8 : Tilting angles for BLFTM, $LaFeO_3$, and BFTM

**Tilt**	**0.72BFTM-0.28LFO $Pmc2_1$**	**0.72BFTM-0.28LFO Pmnb**	**$LaFeO_3$ Pmnb**	**BFTM R3c**
θ $[110]_p$	12.4°	9.79°	10.9°	N/A
ϕ $[100]_p$	10.7°	9.41°	11.0°	N/A
Φ $[111]_p$	16.3°	13.5°	15.4°	11.94°

Figure S4: Cell volume versus composition along the BFTM-LFO line in the phase diagram. The composition refers to the value of x in (x)BFTM-(1-x)LFO

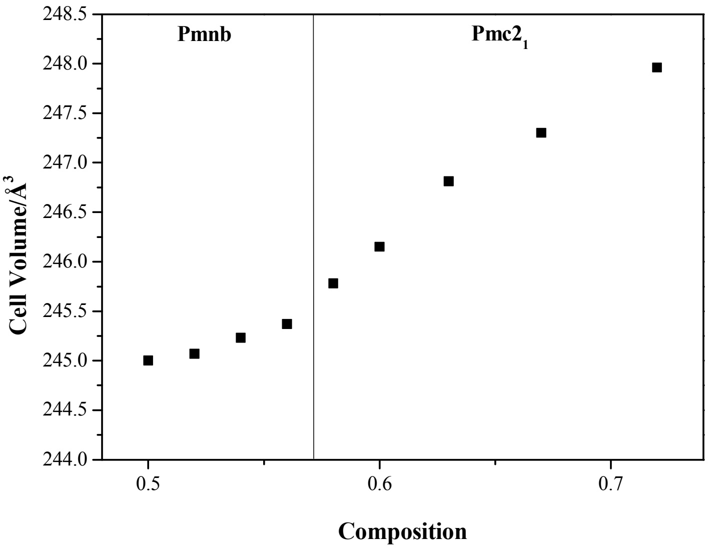

Analysis of the diffraction data from 0.72BFTM-0.28LFO with the programme Platon[3] did not reveal missing symmetry elements, while refinement in lower metric symmetry ($P112_1$ and $Pm11$) did not improve the fit quality. Although the orthorhombic distortion is small, tetragonal symmetry would require the polarisation to lie along the $[001]_p$ direction of the perovskite subcell, and the absence of pseudosymmetry in the missing symmetry analysis shows that this is not the case.

Electron diffraction shows that the new phase adopts a $2a_p x\sqrt{2}a_p x\sqrt{2}a_p$ structure in agreement with the refinements, but due to twinning, it is difficult to determine the reflection conditions. The twinning makes the SAED patterns compositions of patterns from equivalent pseudocubic axes and can be rebuilt according to the refined structure plus twinning. For example, as shown in Figure S4, the SAED pattern $[001]_c$ can be modeled as a superposition of the patterns [100], [011], and [$\bar{1}$00].

Figure S5: : A schematic drawing showing the overlapping effect of diffraction patterns [100], [011] and [$\bar{1}$00] (right) which agree with the highly twinned experimental SAED pattern $[001]_c$ (left).

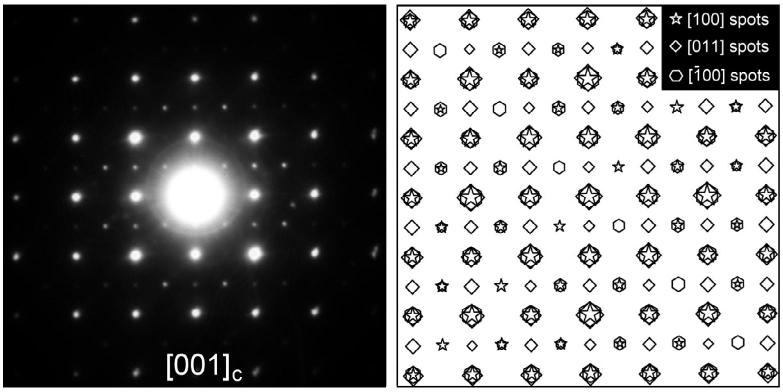

3.2 High Temperature Phase

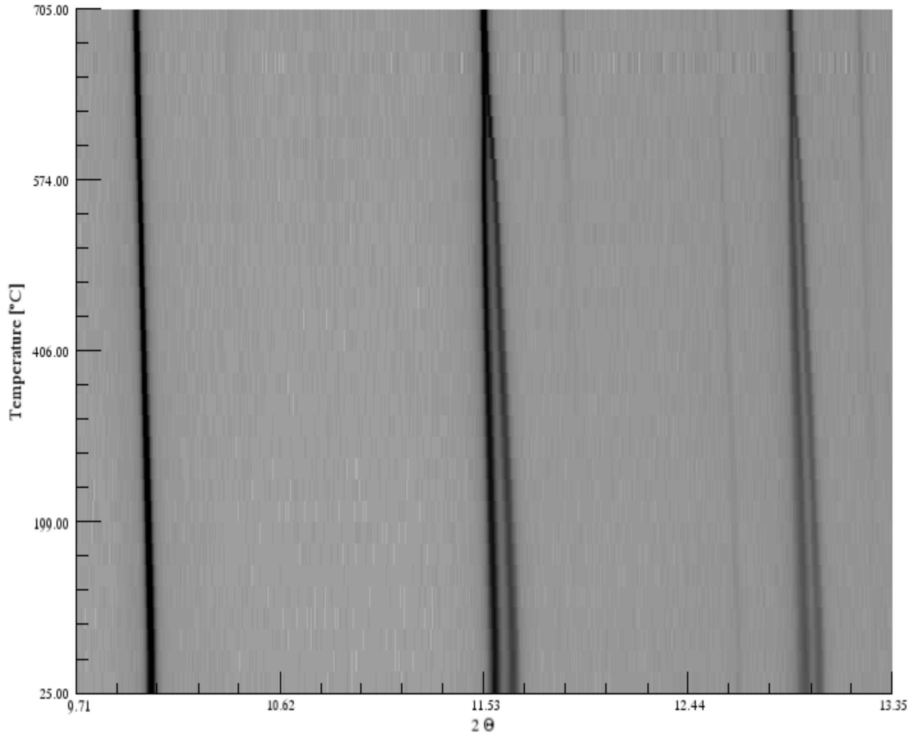

Figure S6: Temperature variation of large *d*-spacing reflections (d=1.72Å-2.36Å) from ID31 XRD diffraction data showing the phase transition from *Pmc2₁* to *Pmnb* above 650°C.

Figure S7: a) Rietveld refinement of 0.72BFTM-0.28LFO from XRD synchrotron data at700°C and b) Rietveld refinement of the same composition from HRPD neutron data at700°C. The unindexed peak in the neutron data at d = 2.2 Å is from the vanadium can used as the sample container during measurements.

a)

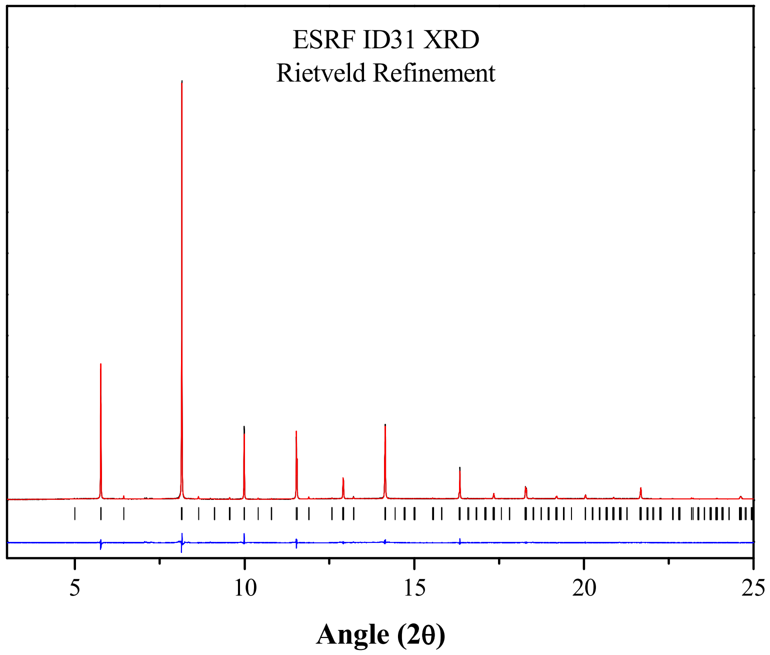

b)

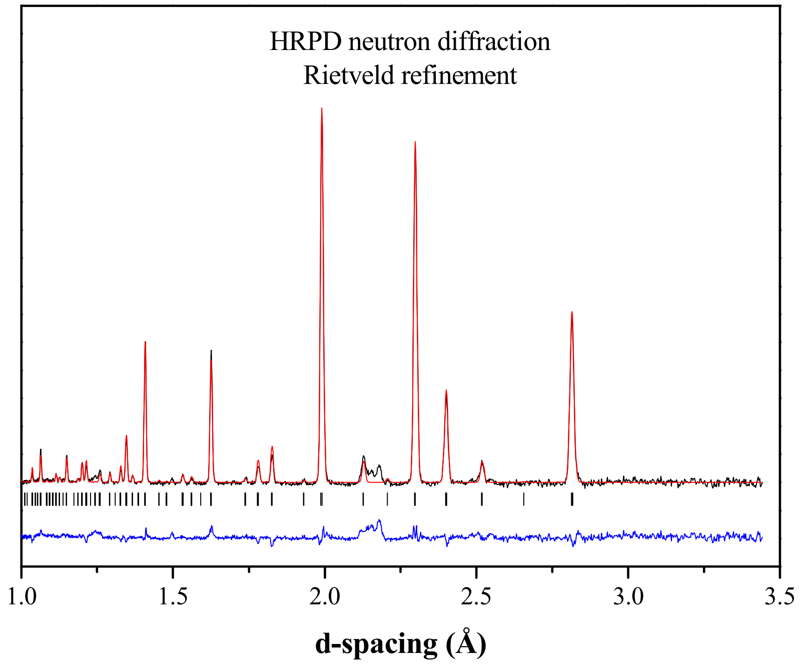

Table S9: Atomic positions from Rietveld refinement of the high temperature *Pmnb* (700 °C) phase of 0.72BFTM-0.28LFO. The lattice parameters are a = 5.63531(3) Å, b = 7.95054(3) Å, c = 5.62813(3) Å.

**Atom**	**x**	**y**	**z**	**Occ**	**B/ Å^2**
Bi/La	039861(1)	0.25	0.5013(4)	0.72/0.28	0.0619(2)
Fe/Mg/Ti	0	0	0	0.46/0.27/0.27	0.0176(3)
O1	0.5031(9)	0.25	0.4339(2)	1	0.045(3)
O2	0.2277(8)	0.4719(9)	0.7201(7)	1	0.040(2)

Table S10: Bond lengths and angles from Rietveld refinement of 0.72BFTM-0.28LFO at 700°C in the *Pmnb* space group.

**Bond**	**Bond Length / Å**
A - O	2.451(2)<br>2 x 2.546(7)<br>2.748(1)<br>2 x 2.773(6)<br>2 x 2.810(7)<br>2.938(1)<br>2 x 3.162(7)<br>3.181(4)
B-O	2 x 1.985(3)<br>2 x 2.022(2)<br>2 x 2.044(3)
**Atoms**	**Angle / °**
O-Bi1/La1/-O	2x55.208(1), 2x56.381(2), 2x56.680(2), 2x58.657(1), 2x58.983(2), 2x60.030(3), 2x60.919(1), 2x61.638(1) 2x62.114(3), 2x 63.777(1), 2x64.312(2), 2x64.759(2), 79.008(1), 80.331(3), 2x84.175(2), 84.295(1), 87.723(3), 88.775(2), 95.703(1), 2x96.084(1),99.672(1), 103.813(3), 2x111.586(1), 2x115.853(2), 2x116.622(2), 2x116.996(2), 2x117.145(2), 2x117.541(1), 2x119.013(1), 2x121.751(3), 2x123.173(3), 2x125.836(2), 2x126.424(1), 2x126.617(3), 164.525(2), 2x171.557(2), 2x174.741(1), 176.034(3)
O-Fe/Mg/Ti-O	2x88.330(3), 2x89.368(1), 2x89.543(1), 2x90.457(2), 2x90.632(1), 2x91.670(1), 3x180.000(2)

Table S11: Bond Valence Sums from high temperature Rietveld refinement of 0.72BFTM-0.28LFO in *Pmnb* at 700°C. The same conclusions regarding local strain and under/over bonding can be made in the *Pmc2₁* and *Pmnb* phases.

Atom	BVS
Bi1	2.1
La1	2.5
Fe	3.0
Ti	3.5
Mg	2.5

3.3 Mode Displacement Analysis

Output from Isodisplace mode analysis for 0.72BFTM-0.28LFO in *Pmc2₁*.

ISODISPLACE: modes details

Parent structure (221 Pm-3m)

```
a=3.94280, b=3.94280, c=3.94280, alpha=90.00000, beta=90.00000, gamma=90.00000
```

atom site x y z occ

```
Bi 1b 0.50000 0.50000 0.50000 0.72000
La 1b 0.50000 0.50000 0.50000 0.28000
Fe 1a 0.00000 0.00000 0.00000 0.47000
Mg 1a 0.00000 0.00000 0.00000 0.27000
Ti 1a 0.00000 0.00000 0.00000 0.27000
O1 3d 0.50000 0.00000 0.00000 1.00000
```

Subgroup details

```
26 Pmc2_1, basis={(2,0,0),(0,1,-1),(0,1,1)}, origin=(1/2,1/2,0), s=4, i=48
```

Undistorted superstructure (26 Pmc2_1)

```
a=7.88560, b=5.57596, c=5.57596, alpha=90.00000, beta=90.00000, gamma=90.00000
```

atom site x y z occ displ

```
Bi1 2a 0.00000 0.25000 0.77920 0.72000 0.00000
Bi2 2b 0.50000 0.25000 0.77920 0.72000 0.00000
La1 2a 0.00000 0.25000 0.77920 0.28000 0.00000
La2 2b 0.50000 0.25000 0.77920 0.28000 0.00000
Fe 4c 0.75000 0.75000 0.77920 0.47000 0.00000
Mg 4c 0.75000 0.75000 0.77920 0.27000 0.00000
Ti 4c 0.75000 0.75000 0.77920 0.27000 0.00000
O1 2a 0.00000 0.25000 0.27920 1.00000 0.00000
O2 2b 0.50000 0.25000 0.27920 1.00000 0.00000
O3 4c 0.25000 0.50000 0.02920 1.00000 0.00000
O4 4c 0.25000 0.00000 0.52920 1.00000 0.00000
```

Distorted superstructure (26 Pmc2_1)

```
a=7.88560, b=5.61276, c=5.61276, alpha=89.98969, beta=90.00000, gamma=90.00000
```

atom site x y z occ displ mag

```
Bi1 2a 0.00000 0.25507 0.76707 0.72000 0.07331 0.00000
Bi2 2b 0.50000 0.25000 0.79133 0.72000 0.06763 0.00000
La1 2a 0.00000 0.25507 0.76707 0.28000 0.07331 0.00000
La2 2b 0.50000 0.25000 0.79133 0.28000 0.06763 0.00000
Fe 4c 0.75000 0.74801 0.79645 0.47000 0.09682 0.00000
Mg 4c 0.75000 0.74801 0.79645 0.27000 0.09682 0.00000
Ti 4c 0.75000 0.74801 0.79645 0.27000 0.09682 0.00000
O1 2a 0.00000 0.17732 0.33885 1.00000 0.52428 0.00000
O2 2b 0.50000 0.32017 0.32028 1.00000 0.45338 0.00000
O3 4c 0.20314 0.50944 0.05915 1.00000 0.40891 0.00000
O4 4c 0.28009 0.03230 0.58060 1.00000 0.41338 0.00000
```

Displacive mode definitions

atom dx dy dz

```
Pm-3m[0,0,0]GM4-(0,a,a)[Bi:b]T1u(a) normfactor = 0.08967
Bi1 0.0000 0.0000 1.0000
Bi2 0.0000 0.0000 1.0000
Pm-3m[0,0,0]GM4-(0,a,a)[La:b]T1u(a) normfactor = 0.08967
La1 0.0000 0.0000 1.0000
La2 0.0000 0.0000 1.0000
Pm-3m[0,0,0]GM4-(0,a,a)[Fe:a]T1u(a) normfactor = 0.08967
Fe 0.0000 0.0000 1.0000
Pm-3m[0,0,0]GM4-(0,a,a)[Mg:a]T1u(a) normfactor = 0.08967
Mg 0.0000 0.0000 1.0000
Pm-3m[0,0,0]GM4-(0,a,a)[Ti:a]T1u(a) normfactor = 0.08967
Ti 0.0000 0.0000 1.0000
Pm-3m[0,0,0]GM4-(0,a,a)[O1:d]A2u(a) normfactor = 0.04484
O1 0.0000 0.0000 0.0000
```

O2 0.0000 0.0000 0.0000
O3 0.0000 -1.0000 1.0000
O4 0.0000 -1.0000 1.0000
Pm-3m[0,0,0]GM4-(0,a,a)[O1:d]Eu(a) normfactor = 0.06341
O1 0.0000 0.0000 1.0000
O2 0.0000 0.0000 1.0000
O3 0.0000 0.5000 0.5000
O4 0.0000 0.5000 0.5000
Pm-3m[0,0,0]GM5-(a,0,-a)[O1:d]Eu(a) normfactor = 0.06341
O1 0.0000 0.0000 -1.0000
O2 0.0000 0.0000 -1.0000
O3 0.0000 0.5000 0.5000
O4 0.0000 0.5000 0.5000
Pm-3m[1/2,1/2,1/2]R4+(a,0,a)[O1:d]Eu(a) normfactor = 0.06341
O1 0.0000 -1.0000 0.0000
O2 0.0000 1.0000 0.0000
O3 -0.5000 0.0000 0.0000
O4 0.5000 0.0000 0.0000
Pm-3m[1/2,1/2,1/2]R5+(a,0,-a)[Bi:b]T1u(a) normfactor = 0.08967
Bi1 0.0000 1.0000 0.0000
Bi2 0.0000 -1.0000 0.0000
Pm-3m[1/2,1/2,1/2]R5+(a,0,-a)[La:b]T1u(a) normfactor = 0.08967
La1 0.0000 1.0000 0.0000
La2 0.0000 -1.0000 0.0000
Pm-3m[1/2,1/2,1/2]R5+(a,0,-a)[O1:d]Eu(a) normfactor = 0.06341
O1 0.0000 -1.0000 0.0000
O2 0.0000 1.0000 0.0000
O3 0.5000 0.0000 0.0000
O4 -0.5000 0.0000 0.0000
Pm-3m[0,1/2,0]X5+(0,0,0,0,0,a)[Bi:b]T1u(a) normfactor = 0.08967
Bi1 0.0000 0.0000 -1.0000
Bi2 0.0000 0.0000 1.0000
Pm-3m[0,1/2,0]X5+(0,0,0,0,0,a)[La:b]T1u(a) normfactor = 0.08967
La1 0.0000 0.0000 -1.0000
La2 0.0000 0.0000 1.0000
Pm-3m[0,1/2,0]X5+(0,0,0,0,0,a)[O1:d]Eu(a) normfactor = 0.08967
O1 0.0000 0.0000 1.0000
O2 0.0000 0.0000 -1.0000
O3 0.0000 0.0000 0.0000
O4 0.0000 0.0000 0.0000
Pm-3m[0,1/2,0]X3-(0,0,a)[Fe:a]T1u(a) normfactor = 0.06341
Fe 1.0000 0.0000 0.0000
Pm-3m[0,1/2,0]X3-(0,0,a)[Mg:a]T1u(a) normfactor = 0.06341
Mg 1.0000 0.0000 0.0000
Pm-3m[0,1/2,0]X3-(0,0,a)[Ti:a]T1u(a) normfactor = 0.06341
Ti 1.0000 0.0000 0.0000
Pm-3m[0,1/2,0]X3-(0,0,a)[O1:d]Eu(a) normfactor = 0.04484
O1 0.0000 0.0000 0.0000
O2 0.0000 0.0000 0.0000
O3 -1.0000 0.0000 0.0000
O4 -1.0000 0.0000 0.0000
Pm-3m[1/2,1/2,0]M2+(0,a,0)[O1:d]A2u(a) normfactor = 0.04484
O1 0.0000 0.0000 0.0000
O2 0.0000 0.0000 0.0000
O3 0.0000 1.0000 -1.0000
O4 0.0000 -1.0000 1.0000
Pm-3m[1/2,1/2,0]M3+(0,a,0)[O1:d]Eu(a) normfactor = 0.04484
O1 0.0000 0.0000 0.0000
O2 0.0000 0.0000 0.0000
O3 0.0000 -1.0000 -1.0000
O4 0.0000 1.0000 1.0000
Pm-3m[1/2,1/2,0]M5-(0,0,0,0,0,a)[Bi:b]T1u(a) normfactor = 0.08967
Bi1 0.0000 -1.0000 0.0000
Bi2 0.0000 -1.0000 0.0000
Pm-3m[1/2,1/2,0]M5-(0,0,0,0,0,a)[La:b]T1u(a) normfactor = 0.08967
La1 0.0000 -1.0000 0.0000
La2 0.0000 -1.0000 0.0000
Pm-3m[1/2,1/2,0]M5-(0,0,0,0,0,a)[Fe:a]T1u(a) normfactor = 0.08967
Fe 0.0000 -1.0000 0.0000
Pm-3m[1/2,1/2,0]M5-(0,0,0,0,0,a)[Mg:a]T1u(a) normfactor = 0.08967
Mg 0.0000 -1.0000 0.0000
Pm-3m[1/2,1/2,0]M5-(0,0,0,0,0,a)[Ti:a]T1u(a) normfactor = 0.08967
Ti 0.0000 -1.0000 0.0000
Pm-3m[1/2,1/2,0]M5-(0,0,0,0,0,a)[O1:d]Eu(a) normfactor = 0.08967
O1 0.0000 -1.0000 0.0000
O2 0.0000 -1.0000 0.0000
O3 0.0000 0.0000 0.0000
O4 0.0000 0.0000 0.0000
Displacive mode amplitudes
mode As Ap dmax
GM4-[Bi:b]T1u(a) 0.00000 0.00000 0.00000
GM4-[La:b]T1u(a) 0.00000 0.00000 0.00000
GM4-[Fe:a]T1u(a) 0.19237 0.09619 0.09618
GM4-[Mg:a]T1u(a) 0.19237 0.09619 0.09618
GM4-[Ti:a]T1u(a) 0.19237 0.09619 0.09618
GM4-[O1:d]A2u(a) 0.22086 0.11043 0.07809
GM4-[O1:d]Eu(a) 0.88248 0.44124 0.31200
GM4- all 0.96880 0.48440
GM5-[O1:d]Eu(a) 0.08816 0.04408 0.03117
GM5- all 0.08816 0.04408
R4+[O1:d]Eu(a) 1.17003 0.58501 0.41367
R4+ all 1.17003 0.58501
R5+[Bi:b]T1u(a) 0.02827 0.01413 0.01413
R5+[La:b]T1u(a) 0.02827 0.01413 0.01413

```
R5+[O1:d]Eu(a) -0.04357 -0.02178 0.01540
R5+ all 0.05913 0.02957
X5+[Bi:b]T1u(a) 0.13527 0.06764 0.06763
X5+[La:b]T1u(a) 0.13527 0.06764 0.06763
X5+[O1:d]Eu(a) 0.10355 0.05178 0.05177
X5+ all 0.21753 0.10876
X3-[Fe:a]T1u(a) 0.00000 0.00000 0.00000
X3-[Mg:a]T1u(a) 0.00000 0.00000 0.00000
X3-[Ti:a]T1u(a) 0.00000 0.00000 0.00000
X3-[O1:d]Eu(a) 0.18702 0.09351 0.06612
X3- all 0.18702 0.09351
M2+[O1:d]A2u(a) -0.00786 -0.00393 0.00278
M2+ all 0.00786 0.00393
M3+[O1:d]Eu(a) 0.24707 0.12354 0.08735
M3+ all 0.24707 0.12354
M5-[Bi:b]T1u(a) -0.02827 -0.01413 0.01413
M5-[La:b]T1u(a) -0.02827 -0.01413 0.01413
M5-[Fe:a]T1u(a) 0.02219 0.01110 0.01109
M5-[Mg:a]T1u(a) 0.02219 0.01110 0.01109
M5-[Ti:a]T1u(a) 0.02219 0.01110 0.01109
M5-[O1:d]Eu(a) 0.01400 0.00700 0.00700
M5- all 0.05720 0.02860
Overall 1.57018 0.78509
```

Figure S8: a) M_3^+ in phase tilting mode and b) the R_5^+ mode with antiferrodistortive A-site displacements along b. Both modes are viewed down the a axis.

a)

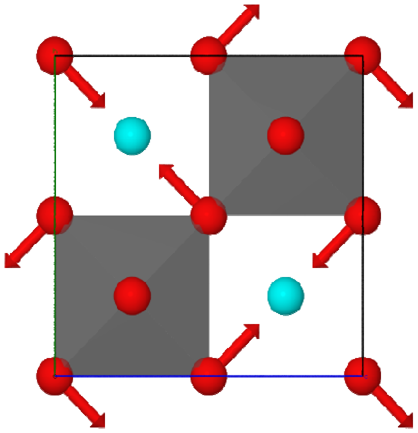

b)

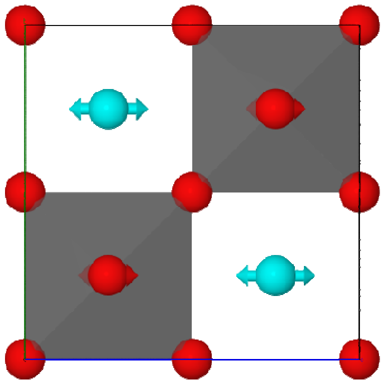

Output from Isodisplace mode analysis for *Pmnb* HT phase of 0.72BFTM-0.28LFO

```
ISODISTORT: modes details
Parent structure (221 Pm-3m)
a=3.94280, b=3.94280, c=3.94280, alpha=90.00000, beta=90.00000, gamma=90.00000
atom site    x        y        z        occ
Bi   1b    0.50000  0.50000  0.50000  0.72000
La   1b    0.50000  0.50000  0.50000  0.28000
Fe   1a    0.00000  0.00000  0.00000  0.46800
Mg   1a    0.00000  0.00000  0.00000  0.27000
Ti   1a    0.00000  0.00000  0.00000  0.27000
O1   3d    0.50000  0.00000  0.00000  1.00000

Subgroup details
62 Pmnb, basis={(2,0,0),(0,1,-1),(0,1,1)}, origin=(0,0,0), s=4, i=24
Undistorted superstructure (62 Pmnb)
a=7.88560, b=5.57596, c=5.57596, alpha=90.00000, beta=90.00000, gamma=90.00000
atom site    x        y        z        occ     displ
Bi   4c    0.25000  0.00000  0.50000  0.72000  0.00000
La   4c    0.25000  0.00000  0.50000  0.28000  0.00000
Fe   4a    0.00000  0.00000  0.00000  0.46800  0.00000
Mg   4a    0.00000  0.00000  0.00000  0.27000  0.00000
Ti   4a    0.00000  0.00000  0.00000  0.27000  0.00000
O1   4c    0.25000  0.50000  0.50000  1.00000  0.00000
O2   8d    0.50000  0.75000  0.75000  1.00000  0.00000

Distorted superstructure (62 Pmnb)
a=7.95050, b=5.63532, c=5.62812, alpha=90.00000, beta=90.00000, gamma=90.00000
atom site    x        y        z        occ     displ
Bi   4c    0.25000  0.01390  0.50130  0.72000  0.07784
La   4c    0.25000  0.01390  0.50130  0.28000  0.07784
Fe   4a    0.00000  0.00000  0.00000  0.46800  0.00000
Mg   4a    0.00000  0.00000  0.00000  0.27000  0.00000
Ti   4a    0.00000  0.00000  0.00000  0.27000  0.00000
O1   4c    0.25000  0.49690  0.43380  1.00000  0.36954
O2   8d    0.47190  0.77230  0.72010  1.00000  0.30390

Displacive mode definitions
 atom    x        y        z        dx      dy      dz
Pm-3m[1/2,1/2,1/2]R4+(a,0,-a)[O1:d]Eu(a) normfactor = 0.06341
O1    0.25000  0.50000  0.50000  0.0000  0.0000 -1.0000
O2    0.50000  0.75000  0.75000 -0.5000  0.0000  0.0000

Pm-3m[1/2,1/2,1/2]R5+(a,0,a)[Bi:b]T1u(a) normfactor = 0.08967
Bi    0.25000  0.00000  0.50000  0.0000  0.0000  1.0000

Pm-3m[1/2,1/2,1/2]R5+(a,0,a)[La:b]T1u(a) normfactor = 0.08967
La    0.25000  0.00000  0.50000  0.0000  0.0000  1.0000

Pm-3m[1/2,1/2,1/2]R5+(a,0,a)[O1:d]Eu(a) normfactor = 0.06341
O1    0.25000  0.50000  0.50000  0.0000  0.0000 -1.0000
O2    0.50000  0.75000  0.75000  0.5000  0.0000  0.0000

Pm-3m[0,1/2,0]X5+(0,0,0,0,a,0)[Bi:b]T1u(a) normfactor = 0.08967
Bi    0.25000  0.00000  0.50000  0.0000 -1.0000  0.0000

Pm-3m[0,1/2,0]X5+(0,0,0,0,a,0)[La:b]T1u(a) normfactor = 0.08967
La    0.25000  0.00000  0.50000  0.0000 -1.0000  0.0000

Pm-3m[0,1/2,0]X5+(0,0,0,0,a,0)[O1:d]Eu(a) normfactor = 0.08967
O1    0.25000  0.50000  0.50000  0.0000  1.0000  0.0000
O2    0.50000  0.75000  0.75000  0.0000  0.0000  0.0000

Pm-3m[1/2,1/2,0]M2+(0,a,0)[O1:d]A2u(a) normfactor = 0.04484
O1    0.25000  0.50000  0.50000  0.0000  0.0000  0.0000
O2    0.50000  0.75000  0.75000  0.0000 -1.0000 -1.0000

Pm-3m[1/2,1/2,0]M3+(0,a,0)[O1:d]Eu(a) normfactor = 0.04484
O1    0.25000  0.50000  0.50000  0.0000  0.0000  0.0000
O2    0.50000  0.75000  0.75000  0.0000  1.0000 -1.0000

Displacive mode amplitudes
 mode              As       Ap      dmax
R4+[O1:d]Eu(a)   0.96520  0.48260  0.34125
R4+  all         0.96520  0.48260

R5+[Bi:b]T1u(a)  0.01450  0.00725  0.00725
R5+[La:b]T1u(a)  0.01450  0.00725  0.00725
R5+[O1:d]Eu(a)   0.07886  0.03943  0.02788
R5+  all         0.08148  0.04074

X5+[Bi:b]T1u(a) -0.15501 -0.07751  0.07750
X5+[La:b]T1u(a) -0.15501 -0.07751  0.07750
X5+[O1:d]Eu(a)  -0.03457 -0.01728  0.01728
X5+  all         0.22193  0.11096

M2+[O1:d]A2u(a)  0.08475  0.04238  0.02996
M2+  all         0.08475  0.04238

M3+[O1:d]Eu(a)   0.58213  0.29107  0.20581
M3+  all         0.58213  0.29107

Overall          1.15480  0.5774
```

4. Properties

4.1 Effect of the addition of MnO_2

As mentioned in the text, MnO_2 is added to lower the dielectric loss. Figure S7 shows the loss of undoped vs doped 0.72BFTM-0.28LFO. The loss of the doped sample is significantly lower, especially at lower frequencies where properties measurements are performed. The power law-like increase in dielectric permittivity of undoped material at low frequencies is reminiscent of hopping conduction.[4] Since this power law-like increase in dielectric permittivity is absent in the Mn-doped material, we suggest that Mn doping prevents electron hopping in the material. Similar decrease of loss upon Mn-doping has been reported for 0.8BiFeO3-0.2BaTiO3 material and has also been ascribed to decrease of electron hopping.[5]

Figure S9: Frequency dependent impedance measurement at room temperature comparing MnO_2-doped to undoped 0.72BFTM-0.28LFO showing a) permittivity and b) loss.

a)

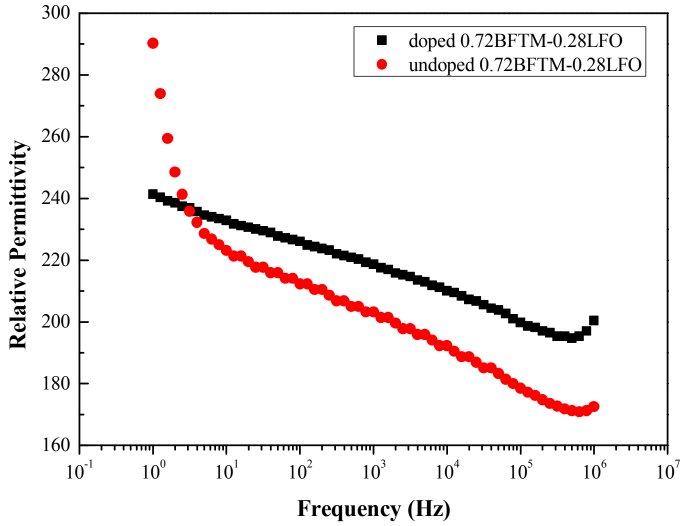

b)

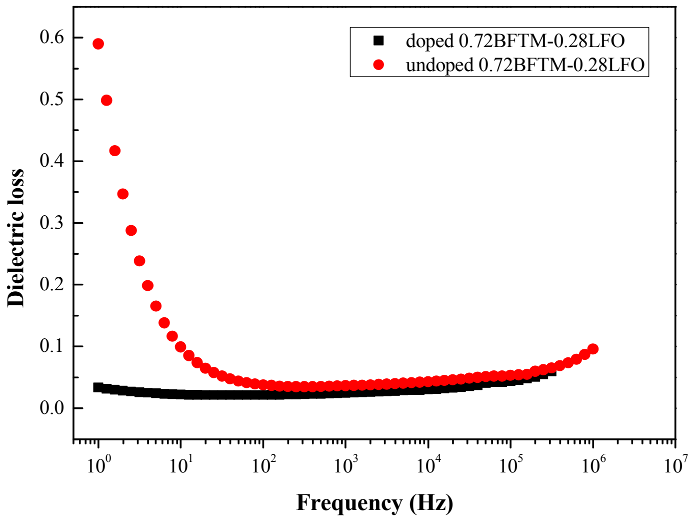

The loss also has an effect on the shape of the P(E) curve as shown in Figure S8 where the undoped sample displays a lossy FE loop and the P(E) loop of the doped sample more closely resembles a typical FE loop.

Figure S10: Example of the P(E) loop for an a) undoped and b) doped sample of 0.625BFTM-0.25LFO-0.125LMT.

a)

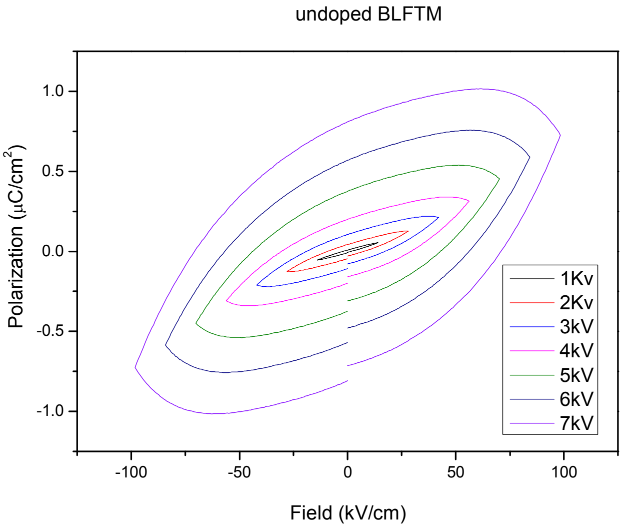

b)

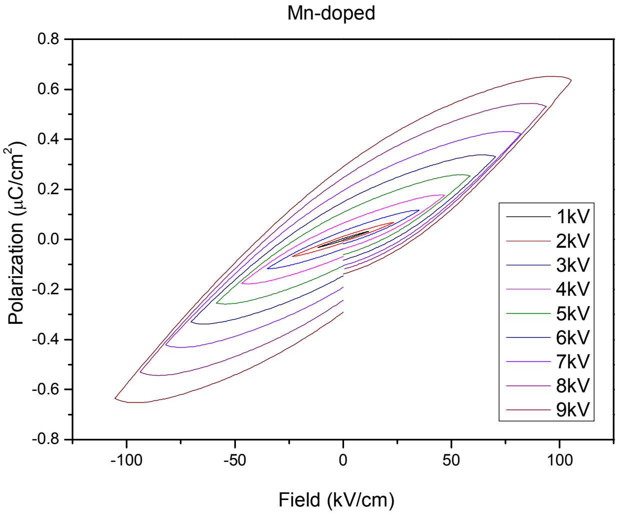

4.2 Poling and d_{33}

For the 0.72BFTM-0.28LFO composition, poling was performed at several different temperatures to determine the optimum condition. The poling electric field and time were fixed at 150kV/cm and 20 minutes respectively. As shown in Table S12, the higher poling temperature gives a better poling effect and higher d_{33} values. Due to the maximum temperature of the silicone oil, the highest temperature we used was 200°C. Once the optimal temperature was found, the sample was poled starting at an electric field 110 kV/cm and the field was increased until the sample broke down.

Table S12: Poling temperature vs d_{33}

**Temperature / °C**	**d_{33} / pC/N**
RT	0.03
50	0.03
100	0.04
150	0.12
200	0.18

Table S13: Electric field vs d_{33}

**Electric field / kV/cm**	**d_{33} / pC/N**
110	0.08
130	0.1
200	0.25

5. Thin film growth and characterisation

0.625BFTM-0.25LFO-0.125LMT thin films were grown by pulsed laser deposition (PLD). All depositions were performed using a PVD Products Nano-PLD system with a 248 nm KrF Lambda Physik excimer laser. A ceramic target was made by conventional solid state methods. (001) oriented 5%Nb-$SrTiO_3$ (Nb-STO) single crystals (PiKem Ltd.) were used as substrates. Substrates were cleaned ultrasonically in acetone and ethanol. The films were grown with laser energy of 195 mJ over a spot size of approximately 3 mm^2, under an oxygen partial pressure of 5 mTorr. The substrate was held at 550 °C and the distance from the target was 50 mm. After depositions were complete, films were held at the growth temperature, and the oxygen pressure was increased to 150 Torr. The films were then cooled at a rate of 10 °C/min under this atmosphere.

For the target, initially, a stoichiometric ceramic 0.625BFTM-0.25LFO-0.125LMT, which gives a composition of $Bi_{0.625}La_{0.375}(Mg_{0.297}Ti_{0.297}Fe_{0.406})O_3$ was used. However, with the above deposition conditions, the PLD process was not stoichiometric, especially for Bi being depleted from the film. Although the process could be improved by modifying deposition conditions, such as increasing laser energy, we still could not obtain the films with expected elemental ratio. Therefore, we prepared a non-stoichiometric ceramic with the composition of $Bi_{0.849}La_{0.317}(Mg_{0.283}Ti_{0.283}Fe_{0.434})O_3$. With this target and the above deposition conditions, the ratio for the obtained films, normalized to Fe was Bi: 1.65(8), La: 0.88(6), Fe: 1.00(9), Ti: 0.74(5), Mg: 0.87(16), which was close to the expected ratio Bi: 1.54, La: 0.92, Fe: 1, Ti: 0.73, Mg: 0.73. It should be mentioned here that the presented composition result was measured with two films by EDS (as described in Supplementary Information 2). The A site element (Bi, La) ratio was determined by measuring Bi, La and Fe contents in the film deposited with normal conditions (Nb-STO substrate at 550 °C). In order to measure the B site element (Mg, Ti and Fe) ratio, the effect of Ti in Nb-STO should be eliminated. So a film was deposited on an Si substrate at room temperature. The change of substrate and deposition temperature will not affect the content of Mg, Ti and Fe, as none of them are volatile.

X-ray diffraction data were collected on a PANalytical X'Pert diffractometer with Cu $K\alpha_1$ radiation (λ = 1.5406 Å). The result for a 200 nm film is shown in Figure S9 (a). As a reference, Figure S9 (b) shows the result of the 0.625BFTM-0.25LFO-0.125LMT powder sample. A series of (00*l*) reflections for the 0.625BFTM-0.25LFO-0.125LMT film confirms the highly oriented growth for the film. The out-of-plane pseudocubic a_p parameter was found to be 3.976 Å. The powder sample shows the orthorhombic phase with lattice parameters a = 7.887Å, b = 5.597Å and c = 5.595Å, giving a calculated pseudocubic a_p of 3.953Å. The consistency in cell parameters between the thin film and powder sample gives the evidence that the film has similar composition to 0.625BFTM-0.25LFO-0.125LMT powder sample, which is also consistent with the above EDS result.

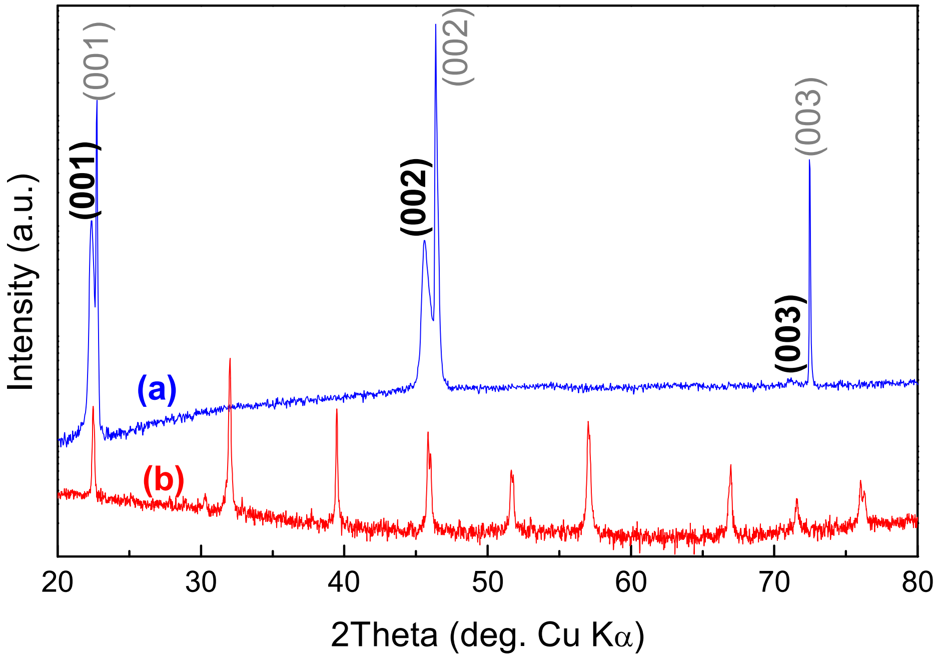

Figure S11: $\theta/2\theta$ XRD scan of (a) a 200 nm 0.625BFTM-0.25LFO-0.125LMT film indexed to the perovskite pseudocubic cell (gray indices indicate a reflection from the Nb-doped $SrTiO_3$ substrate) and (b) 0.625BFTM-0.25LFO-0.125LMT powder.

[1] A. A. Coelho, *Journal of Applied Crystallography* **2000**, *33*, 899.
[2] a)A. C. Larson, R. B. Von Dreele, *Los Alamos National Laboratory Report* **1994**, *LAUR 86-748*; b)B. H. Toby, *Journal of Applied Crystallography* **2001**, *34*, 210.
[3] A. L. Spek, Utrect University, Utrecht, The Netherlands, **2002**.
[4] P. Lunkenheimer, S. Krohn, S. Riegg, S. Ebbinhaus, A. Reller, A. Loidl, *European Physical Journal - Special Topics* **2010**, *180*, 61.
[5] N. Itoh, T. Shimura, W. Sakamoto, T. Yogo, *Ferroelectrics* **2007**, *356*, 19.
